# A pilot study of rivastigmine in the treatment of delirium after stroke: A safe alternative

**DOI:** 10.1186/1471-2377-8-34

**Published:** 2008-09-20

**Authors:** Annemarie W Oldenbeuving, Paul LM de Kort, Ben PW Jansen, L Jaap Kappelle, Gerwin Roks

**Affiliations:** 1 Department of neurology, St Elisabeth Hospital, Tilburg, the netherlands; 2 Department of neurology, TweeSteden Hospital, Tilburg, the netherlands; 3 Department of neurology, University Medical Center, Utrecht, the netherlands

## Abstract

**Background:**

Delirium is a common disorder in the early phase of stroke. Given the presumed cholinergic deficiency in delirium, we tested treatment with the acetylcholinesterase inhibitor rivastigmine.

**Methods:**

This pilot study was performed within an epidemiological study. In 527 consecutive stroke patients presence of delirium was assessed during the first week with the confusion assessment method. Severity was scored with the delirium rating scale (DRS). Sixty-two patients developed a delirium in the acute phase of stroke. Only patients with a severe and persistent delirium (defined as a DRS of 12 or more for more than 24 hours) were enrolled in the present study. In total 26 fulfilled these criteria of whom 17 were treated with orally administered rivastigmine with a total dose between 3 and 12 mg a day. Eight patients could not be treated because of dysphagia and one because of early discharge.

**Results:**

No major side effects were recorded. In 16 patients there was a considerable decrease in severity of delirium. The mean DRS declined from 14.8 on day one to 8.5 after therapy and 5.6 after tapering. The mean duration of delirium was 6.7 days (range; 2–17).

**Conclusion:**

Rivastigmine is safe in stroke patients with delirium even after rapid titration. In the majority of patients the delirium improved after treatment. A randomized controlled trial is needed to establish the usefulness of rivastigmine in delirium after stroke.

**Trial registration:**

Nederlands Trial Register NTR1395

## Background

Delirium is a frequent complication of stroke and sometimes the presenting feature. Estimates of the incidence of delirium in the early phase of stroke range from 13 to 48% dependent on study population and delirium definition. [1-6] Given the longer hospitalization period and poorer prognosis, adequate treatment is important[3,4]. Sedative and anti-psychotic drugs are frequently used, but the effect of these drugs in stroke patients is often disappointing and severe side effects are reported[7].

Decreased cholinergic activity,[8,9] decreased cholinergic reserve, and decreased acetylcholine in the basal nucleus of Meynert [10] are postulated as neurochemical correlates of delirium. Furthermore, drugs with anticholinergic effects may induce delirium,[11] and cholinergic drugs can improve delirium induced by lithium and anticholinergic medication.[12,13] Therefore, treating the cholinergic deficiency in delirium patients might be beneficial. Acetylcholinesterase inhibitors have been used successfully in the treatment of patients with Alzheimer's disease [14] and Lewy body dementia.[15]

To the best of our knowledge cholinergic drugs have not been studied in patients with delirium in the early phase post-stroke. In the present study we aimed to assess the feasibility and safety profile of the acetylcholinesterase inhibitor rivastigmine in patients with delirium after a recent stroke. Because of the need for rapid intervention in delirium we tested a novel titration scheme for rivastigmine.

## Methods

In a prospective study, 527 consecutive stroke patients were screened for the presence of delirium by means of the Confusion Assessment Method (CAM) during the first week of admission[16]. If positive, the severity was measured daily with the Delirium Rating Scale (DRS)[17]. Screening for delirium was performed twice, on day 2–4 and day 5–7. In case of delirium the DRS was repeated daily. Patients with a severe and persistent delirium, defined as a DRS above 12 for more than 24 hours,[17] were included in the current pilot study. Sufficient effect of treatment was defined as a DRS of 10 or less. Pre-existing cognitive decline was measured by means of the Informant Questionnaire on Cognitive Decline in the Elderly (IQCODE).[18,19] Stroke subtype and severity were scored with the Oxfordshire Community Stroke Project (OCSP) criteria [20,21] and the National Institutes of Health Stroke Scale (NIHSS).[22].

Rivastigmine was started in a dose of 1.5 mg b.i.d., and was raised every other day by 3 mg, depending on the clinical response, with a maximum of 12 mg a day (i.e. day 1: 1.5 mg b.i.d., day 3: 3 mg b.i.d., day 5: 4.5 mg b.i.d., day 7: 6 mg b.i.d.). If the DRS remained below 10 the rivastigmine dosage was continued for one week and subsequently tapered at a rate similar to the titration scheme.

The study protocol was reviewed and approved by the medical ethics committee of the St Elisabeth Hospital Tilburg (ref 0307) and subsequently approved in a short procedure by the medical ethics committee of the TweeSteden Hospital Tilburg. This study was carried out in compliance with the Helsinki Declaration. Informed consent was obtained from the caregivers.

## Results

In the prospective study 62 patients (11.6%) had a delirium. Thirty-four of these patients had a DRS score below twelve, and 2 were already treated with antipsychotic medication. Hence, 26 patients were included in the present study. Since at time of the study rivastigmine could be administered by oral route only, it had to be interrupted in eight patients with severe dysphagia. In one patient rivastigmine was stopped because of discharge to a nursing home. Characteristics and treatment effect of the 17 patients included in the study are summarized in Table 1. The mean age of the treated patients was 77 years, 65% were men. Right sided hemispheric strokes occurred in 59%. The median NIHSS on admission was 9 (range 2–32).

**Table 1:** Patient characteristics and treatment effect of rivastigmine.

**Patient (gender)**	**Age (years)**	**IQCODE**	**Stroke localization**	**Stroke type**	**NIHSS**	**Highest daily dose (mg)**	**Effect**	**Delirium duration (days)**
1(F)	69.2	53	Right	PACI	5	6	Yes	4
2(M)	73.9	48	Right	TACI	15	9	Yes	6
3(M)	85.0	70	Right	POCI	3	3	Yes	3
4(M)	83.4	48	Right	POCI	6	9	Yes	11
5(M)	80.7	48	Right	PACI	4	6	Yes	3
6(F)	74.1	48	Right	PACI	3	3	Yes	2
7(M)	53.4	48	Left	PACI	16	12	Yes	14
8(M)	75.0	48	Right	TACI	11	12	Yes	17
9(M)	82.5	48	Right	POCI	4	3	Yes	2
10(F)	78.2	48	Right	PACI	2	9	Yes	11
11(M)	85.5	58	Right	LACI	3	3	Yes	2
12(M)	72.9	51	Left	TACI	20	6	Yes	5
13(F)	72.8	78	Left	ICH	9	9	No	14
14(M)	86.5	48	Left	LACI	7	9	Yes	8
15(F)	79.3	48	Left	PACI	9	6	Yes	4
16(M)	76.6	48	Left	ICH	15	6	Yes	8
17(F)	79.4	48	Left	ICH	9	9	Yes	7

F = Female; M = Male. PACI = Partial Anterior Circulation Infarction; TACI = Total Anterior Circulation Infarction; POCI = Posterior Circulation Infarction; LACI = Lacunar Infarction; ICH = Intracerebral Hemorrhage

**Figure 1:**
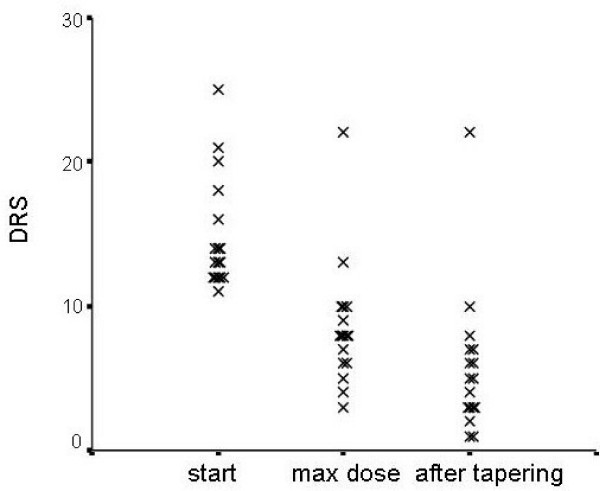
**DRS values according to treatment status**. Figure one shows the course of the DRS, visualized at three points. Every patient is shown with an X. The first point is the DRS the day rivastigmine is started, the second is at maximum dose of rivastigmine and the last is after tapering off rivastigmine.

Of the 17 patients with delirium, 12 had an infection and were treated with antibiotics. Seven patients had a metabolic disturbance that could causally be related to the delirium. Nine patients had a hyperactive/hyperalert type of delirium, 6 hypoactive/hypoalert, and 2 had a mixed type. In 16 of the 17 (94%) patients there was a decrease in severity of delirium following rivastigmine treatment (Figure 1). The mean DRS decreased from 14.8 at start of rivastigmine to 8.5 at individual maximum dose and 5.6 after tapering. Four patients needed 3 mg rivastigmine per day, 5 patients 6 mg, 5 patients 9 mg, and 2 patients needed 12 mg. The mean duration of the delirium in these 16 patients was 6.7 days (range 2–17). In all patients it was possible to taper the dose of rivastigmine without recurrence of delirium in the first month. One patient showed no response at all, despite a daily rivastigmine dose of 9 mg (patient 13). This patient was admitted with an acute confusion that was caused by an intracerebral hemorrhage. Her medical history reported dementia but she was never formally analyzed and diagnosed. The IQCODE of 78 was consistent with dementia. When treating with rivastigmine there was no effect at all and despite the fact that she was not at the highest dose we chose to change her medication to haloperidol. The confusion only slightly declined but since there was no aggression no further sedative drugs were started. After 2 weeks she was admitted to a nursing home and was lost to follow up.

Only 2 patients suffered from diarrhoea (both at high doses), which in one of them could be caused by a pre-existing ulcerative colitis. Nausea or cardiac complications did not occur. Five patients needed additional medication because of agitation at night and insomnia. These patients were treated with temazepam in a dose between 10–20 mg. Haloperidol was allowed in the protocol as rescue medication. Only in one patient (pat 13) this has been used.

## Discussion

We showed that it is possible and safe to treat patients with severe delirium in the acute phase after stroke with rivastigmine, and that low doses sufficed in the majority of the patients. This is in line with the excellent tolerability in Lewy body and Parkinson dementia, diseases with a presumed severe cholinergic deficiency.[15,23]. An important observation is the possibility to increase the dose rapidly without serious side effects making rapid intervention possible. A major drawback in the use of rivastigmine was the absence of a parenteral route for administration. In confused patients unable to swallow, nasogastric feeding is often not tolerated or unsafe.

In the American practice guideline for treatment of delirium, haloperidol is first choice,[24] notwithstanding its anticholinergic side-effects,[8] a risk factor for delirium.[11] Moreover, haloperidol may interfere with recovery after stroke and therefore should be avoided if possible.[25] A possible explanation for the effect of rivastigmine can be an improvement of preexisting dementia-symptoms in our patient group. However, in the medical history only 1 patient was reported with dementia (patient 13). We did not use the minimal mental state examination (MMSE) as an indicator of dementia because it has been shown to be unreliable in the acute phase after stroke.[26] Instead we used the IQCODE which gives an impression of cognitive deterioration in the past ten years. Using the cut off point of 3.9 per item [27] only 2 patients were in the range of dementia (patient 3 and 13). Also the fact that we could taper the dose of rivastigmine without recurrence of the cognitive symptoms indicates that we indeed treated the delirium symptoms and not the symptoms of undiagnosed dementia. Case reports have suggested that acetylcholinesterase inhibitors can be used in the treatment of delirium in patients with dementia, Parkinson's disease, and elderly patients with long-standing delirium.[12,13,28] Treatment with rivastigmine has not been described in patients with delirium in the early phase after stroke. Obviously, the uncontrolled design and small number of patients do not allow conclusions with respect to the therapeutic value in patients with delirium after stroke, but our study suggests that rivastigmine might be useful in this setting. Therefore, a randomized controlled trial is needed in which rivastigmine will be compared with standard treatment. The novel transdermal administration of rivastigmine may be indicated in patients with delirium after stroke because of frequent swallowing problems[29].

## Conclusion

Rivastigmine is safe in stroke patients with delirium even after rapid titration. In the majority of patients the delirium improved after treatment. A randomized controlled trial is needed to establish the usefulness of rivastigmine in delirium after stroke.

## Abbreviations

CAM: Confusion Assessment Method; DRS: Delirium Rating Scale; IQCODE: Informant Questionnaire on Cognitive Decline in the Elderly; OCSP: Oxfordshire Community Stroke Project; NIHSS: National Institutes of Health Stroke Scale; MMSE: Minimal mental state examination.

## Competing interests

Part of our further research on delirium is supported by Novartis. Novartis had no role in the design, execution, analysis and interpretation of data, or writing of the article.

## Authors' contributions

AO participated in data collection, coordinated the study and helped to draft the manuscript. PLMK participated in data collection, study design and helped to draft the manuscript.  BPWJ participated in data collection, study design and helped to draft the manuscript. LJK participated in the study design and helped to draft the manuscript. GR participated in data collection, study design and helped to draft the manuscript. All authors read and approved the final manuscript.

## Pre-publication history

The pre-publication history for this paper can be accessed here:


